# Parental and sexual conflicts over the *Peg3* imprinted domain

**DOI:** 10.1038/srep38136

**Published:** 2016-11-30

**Authors:** Hongzhi He, Bambarendage P. U. Perera, An Ye, Joomyeong Kim

**Affiliations:** 1 Department of Biological Sciences, Louisiana State University, Baton Rouge, LA 70803, USA

## Abstract

In the current study, the imprinting control region of the mouse *Peg3* domain was deleted to test its functional impact on animal growth and survival. The paternal transmission of the deletion resulted in complete abolition of the transcription of two paternally expressed genes, *Peg3* and *Usp29*, causing the reduced body weight of the pups. In contrast, the maternal transmission resulted in the unexpected transcriptional up-regulation of the remaining paternal allele of both *Peg3* and *Usp29*, causing the increased body weight and survival rates. Thus, the imprinted maternal allele of the ICR may be a suppressor antagonistic to the active paternal allele of the ICR, suggesting a potential intralocus allelic conflict. The opposite outcomes between the two transmissions also justify the functional compromise that the maternal allele has become epigenetically repressed rather than genetically deleted during mammalian evolution. The mice homozygous for the deletion develop normally but with a skewed sex ratio, one male per litter, revealing its sex-biased effect. Overall, the *Peg3* locus may have evolved to an imprinted domain to cope with both parental and sexual conflicts driven by its growth-stimulating paternal versus growth-suppressing maternal alleles.

*Peg3* (Paternally Expressed Gene 3) encodes a zinc finger protein that controls its downstream targets as a transcriptional repressor^1^,^2^. *Peg3* is also known to control the fetal growth rates and maternal-caring behaviors of placental mammals^3^,^4^,^5^. In particular, the mutant pups tend to have a problem in suckling milk whereas mutant females have defects in the milk letdown process^5^. Although these observations have been recently refuted by an independent study^6^, these phenotypes suggest potential roles of *Peg3* in the oxytocin circuitry^5^. Consistent with this, *Peg3* is highly expressed in embryos and placentas as well as the tissues involved in the oxytocin circuitry, including the hypothalamus, uterus and mammary gland^7^,^8^,^9^. Genome-wide expression analyses indicated that several placenta-specific gene families are up-regulated in the mutant embryos^4^. The up-regulation observed from these female-biased gene families further hints at potential sex-specific roles of *Peg3*. As seen in the other sex-specific genes, the expression levels of *Peg3* itself are sexually biased with males displaying two-fold higher levels than females^10^. Independent observations also confirmed that several mutations on the *Peg3* locus often cause sex-biased outcomes^11^,^12^. Thus, it has been thought that the *Peg3* locus may be subject to unknown regulatory mechanisms that have been optimized for the different demands from the two sexes during mammalian evolution.

*Peg3* is also subject to an unusual epigenetic mechanism, genomic imprinting, by which the maternal allele is repressed by DNA methylation during oogenesis^13^,^14^. Thus, *Peg3* is expressed mainly from the paternal allele^7^. Placental mammals are hypothesized to have parental conflicts over the gene dosage of a subset of loci that are critical for the survival and growth of their offspring^15^,^16^. As such, the paternally expressed genes are growth-stimulators whereas the maternally expressed genes are growth-suppressors. Consistent with this, several targeted mutations on the *Peg3* locus indeed resulted in smaller body sizes, confirming the paternally expressed *Peg3* as a growth stimulator^3^,^4^. Thus, genomic imprinting may have been set up on the *Peg3* locus as an outcome of this parental conflict during mammalian evolution. *Peg3* is also surrounded by 6 additional imprinted genes that are localized within a 500-kb genomic interval, including paternally expressed *Usp29, Zfp264, APeg3* and maternally expressed *Zim1, Zim2, Zim3*^17^. This imprinted domain, termed the *Peg3* domain, has been well conserved during evolution^17^. The *in vivo* functions of the other imprinted genes, however, have not been well characterized so far. Nevertheless, *Peg3* is likely the most critical gene in terms of controlling fetal growth rates and other related functions given the frequent loss of the open reading frames in the other imprinted genes during evolution^17^,^18^.

As seen in the other imprinted domains, the *Peg3* domain is also regulated through an imprinting control region (ICR), which encompasses the bidirectional promoter of two paternally expressed genes, *Peg3* and *Usp29*^11^. As part of ongoing efforts, we have deleted the entire region of this ICR in the current study. As expected, this deletion resulted in disruption of the imprinting of several genes. Unexpectedly, however, this deletion also provided several unique observations that have not been seen in the other ICRs. Deletion of the seemingly inactive maternal allele resulted in the transcriptional up-regulation of the remaining active paternal allele of *Peg3* and *Usp29*, causing the increased survival and growth rates among the mutant pups. Thus, the imprinted maternal allele of the ICR may be a suppressor influencing the activity of the remaining paternal allele. Detailed results along with relevant discussion are described below.

## Results

### Generation of a mutant mouse line deleting the Peg3-DMR

**Figure 1:**
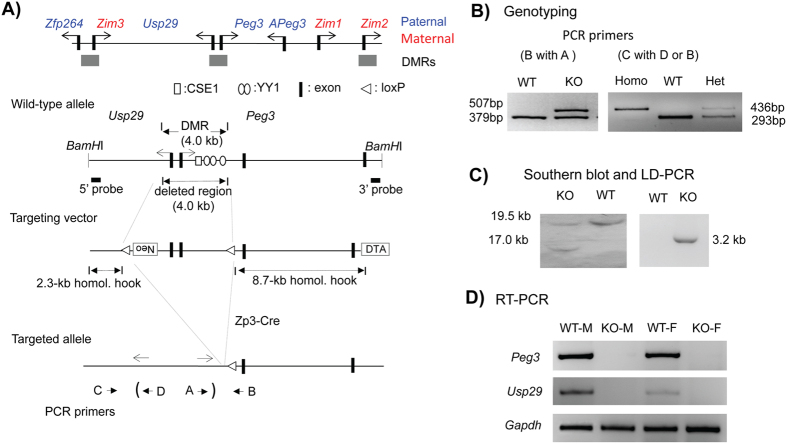
*Peg3* domain and the KO scheme. (**A**) Schematic representation of the *Peg3* domain (upper panel). Each imprinted gene is indicated with an arrow. The paternally and maternally expressed genes are indicated with blue and red, respectively. The three DMRs are indicated with gray boxes. Targeting scheme (lower panel). The 4.0-kb Peg3-DMR contains the first exons of *Peg3* and *Usp29* and the 2.5-kb YY1 binding region. The transcriptional direction of *Peg3* and *Usp29* is indicated with arrows, and exons are indicated with thick vertical lines. The region corresponding to the neomycin resistance gene (*NeoR*) along with the two flanking loxP sites within the targeting vector are indicated by an open box and triangles, respectively. Arrows underneath ‘Targeted allele’ indicate primers with relative positions that were used for PCR-based genotyping. (**B**) PCR-based genotyping. Two combinations of three primers (left panel: B with A) and (right panel: C with either D or B) were used for testing the proper deletion of the 4-kb Peg3-DMR. Two primers (**D** and **A**) are localized within the deleted region, thus indicated with parentheses. The sizes for WT and KO alleles in the downstream loxP are 379 and 507 bp in length, whereas those for the upstream loxP site are 293 and 436 bp in length. (**C**) Southern blot and Long-Distance PCR. *BamH*I-digested DNA from WT and KO were probed with the 3′-side probe, showing WT and KO alleles with different sizes. LD-PCR was also performed to monitor the 5′-side of recombination using a set of primers. (**D**) RT-PCR confirming abolition of the transcription of *Peg3* and *Usp29*. The total RNA from the heads of one-day-old neonates with WT and KO with the paternal transmission was used for this series of RT-PCR analyses. Due to the deletion of the bidirectional promoter for *Peg3* and *Usp29*, the expression of both genes was not detectable at all in the KO samples of both sexes. *Gapdh* was used as a loading control.

In the *Peg3* domain, the 4-kb genomic region encompassing the 1.5-kb bidirectional promoter of *Peg3* and *Usp29* and the 2.5-kb 1^st^ intron of *Peg3* with multiple YY1 binding sites is differentially methylated between two alleles (Fig. 1A). This DMR has been predicted to be an ICR for this domain, and subsequently confirmed through the previous study deleting the 2.5-kb YY1 binding region^11^. In the current study, the entire 4-kb region was targeted again with a slightly different scheme. The 4-kb DMR was flanked with two loxP sites, allowing the deletion of this region with the Cre recombinase (Fig. 1A,C). The detailed information regarding this floxed allele has been recently published^19^,^20^. For the current study, this mouse line with this floxed allele was crossed with the Zp3-Cre line, subsequently generating the mutant line lacking the 4-kb DMR region. PCR-based assays using two sets of primers indeed confirmed the proper deletion of the 4-kb region (Fig. 1B). Since the deleted region contains the bidirectional promoter of the two paternally expressed genes, *Peg3* and *Usp29*, the expression of these two imprinted genes were also tested using the total RNA isolated from the brains of one-day-old neonates with the paternal transmission of the deleted allele. As shown in Fig. 1D, the expression of these two genes was not detectable at all, confirming complete abolition of the transcription of *Peg3* and *Usp29*. This also confirmed faithful silencing of the transcription of the remaining maternal alleles by genomic imprinting. Overall, the results confirmed the successful generation of the mutant mouse line lacking the Peg3-DMR.

### Mutational effects on the survival and growth rates of the animals

**Figure 2:**
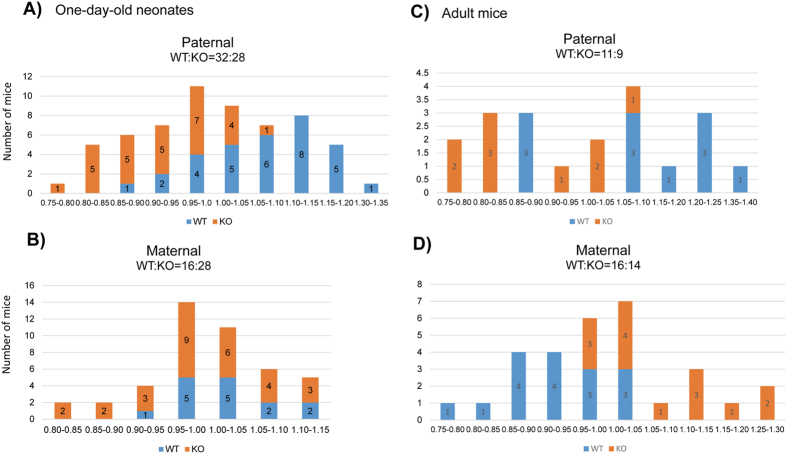
Mutational effects on survival and growth rates. Male and female heterozygotes for the deletion of the Peg3-DMR were individually bred with their wild-type littermates for the paternal and maternal transmission of the deleted allele. The body weight of each pup was first divided by the average weight of a given litter, providing a percentile score for each pup. The values on the X axis indicate these percentiles, while the values on the Y axis indicate the number of mice (**A**,**B**). A similar series of analyses were also performed using the two sets of weaned mice of two-month-old age with the paternal and maternal transmission of the deleted allele (**C**,**D**).

The deletion effects of the Peg3-DMR on the survival and growth rates of the animals were analyzed using the following breeding schemes. First, the males and females heterozygous for the deletion were individually crossed with their female and male littermates, deriving the pups with the paternal and maternal transmission of the deletion, respectively (Fig. 2A,B). The sex and genotype of one-day-old pups were recorded, and also the health status was monitored through measuring their birth weights (Supplemental material 1). The weight of each pup was further normalized by the average weight of a given litter, and subsequently used for generating the weight profiles of a given genotype. Careful inspection of the results provided the following conclusions. The paternal transmission of the deletion did not cause any embryonic lethality based on the litter sizes (60 pups/7 litters = 8.57) and transmission ratio (WT:KO = 32:28) (Fig. 2A). However, the paternal transmission resulted in a dramatic reduction in the body weight of the pups: the average body weights in percentage were 107.1% in WT versus 91.9% KO (Student’s t-test, *p* = 0.0001). This agrees with the previous observations that the loss-of-function-type mutations on the *Peg3* locus usually cause the reduced body weight among the animals^3^,^4^,^11^. In contrast, the maternal transmission caused no major difference in the weight profile of the neonates between WT and KO (Fig. 2B). Interestingly, however, the transmission ratio was somewhat skewed more towards the direction of favoring the KO allele (WT:KO = 16:28) (chi-square test, *p* = 0.0704), although the litter size (44/5 = 8.8) was within the normal range. This suggests that the KO embryos may have outcompeted the WT embryos before the implantation stage. Second, the weaned mice were also similarly analyzed using their weight profiles (Fig. 2C,D). The paternal transmission again resulted in a dramatic reduction of the adult weight, 109.6% in WT versus 89.3% in KO (Student’s t-test, *p* = 0.0001), which is consistent with the pattern seen in the neonates. On the other hand, the maternal transmission resulted in a dramatic increase in the body weight of the adult mutant mice, 92.3% in WT versus 108.3% in KO (Student’s t-test, *p* = 0.0001). This is also consistent with the results from the neonates that the KO mice were more survivable than the WT mice during embryogenesis. Overall, this series of breeding experiments concluded that the paternal transmission of the deletion resulted in the reduced body weight during both neonatal and postnatal stages, and that the maternal transmission caused increased survival rates during the embryonic stage and increased body weights during the postnatal stage.

### Mutational effects on the imprinting status of the *Peg3* domain

**Figure 3:**
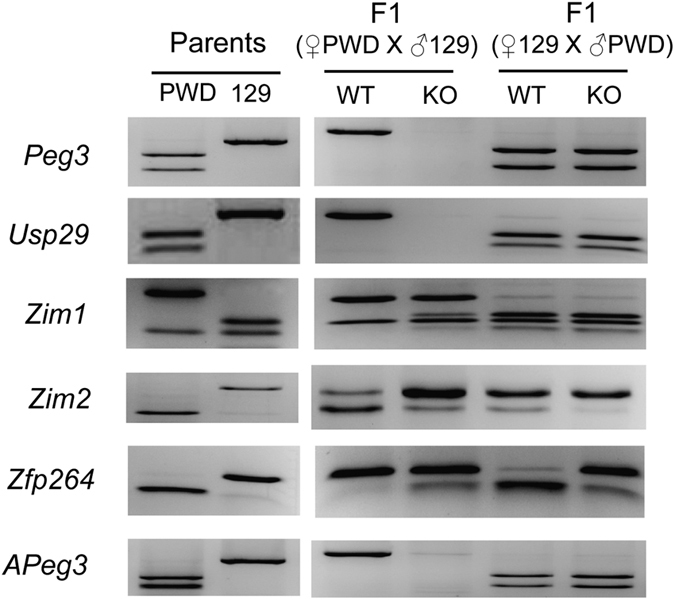
Mutational effects on the imprinting status of the *Peg3* domain. RT-PCR-based imprinting test of the genes within the *Peg3* domain. This series of imprinting tests used the total RNA isolated from the neonatal heads of the F1 hybrid of the male set that had been prepared through the reciprocal crossing of the heterozygotes with the 129/B6 background and the breeding partners with the PWD/PhJ background. The products from RT-PCR were digested with a given restriction enzyme to differentiate parental alleles, which are shown as different-size DNA fragments on gel images. The two columns on the left represent the digestion patterns for two parental strains for each gene; the two middle columns represent the results from the F1 hydrid set with the paternal transmission of the KO allele (male heterozygote with female PWD/PhJ); the two columns on the right represent the results from the F1 hybrid set with the maternal transmission of the KO allele (male PWD/PhJ with female heterozygote). The original gel image for each gene is available through Supplemental Information.

The deletion effects on the imprinting status of the *Peg3* domain were analyzed using the total RNA isolated from the neonatal brains of male F1 hybrids that had been prepared through the reciprocal crossing of the KO animals of the 129/B6 background and the WT animals of the PWD/PhJ background (Fig. 3). The results from the female set are also presented in Supplemental material 2. The results are summarized as follows. First, the deletion effects through the paternal transmission on the two genes, *Peg3* and *Usp29*, were not measurable since there was no detectable expression in either case. The maternal transmission did not cause any change in the imprinting status of these two paternally expressed genes, either. Second, the imprinting status of *Zim1* was not affected at all by both transmissions, confirming that the imprinting of *Zim1* is unlikely controlled through the Peg3-DMR. A similar outcome was also observed from the previous study deleting only the 2.5-kb YY1 binding region^11^. Third, the imprinting status of the two adjacent genes, *Zim2* and *Zfp264*, was affected the most dramatically by the deletion of the Peg3-DMR. In the paternal transmission, the maternally expressed *Zim2* became a paternally expressed gene. In the maternal transmission, there was no major change in the maternal expression status of *Zim2*. In the case of *Zfp264*, interestingly, its paternal expression became biallelic in both transmissions. Fourth, the imprinting status of *APge3* was not affected in both transmissions. However, the expression levels of this antisense gene were reduced dramatically in the paternal transmission, 10-fold lower than the levels of the wild type (data not shown), suggesting that the Peg3-DMR may be involved in controlling the transcriptional levels of *APeg3*. The DNA methylation levels of the DMRs were also analyzed and presented as Supplemental material 3. In summary, this series of analyses concluded that the imprinting of the two adjacent genes, *Zim2* and *Zfp264*, were affected the most by the deletion of the Peg3-DMR, confirming the ICR roles of the Peg3-DMR.

### Mutational effects on the expressional levels of the *Peg3* domain

**Figure 4:**
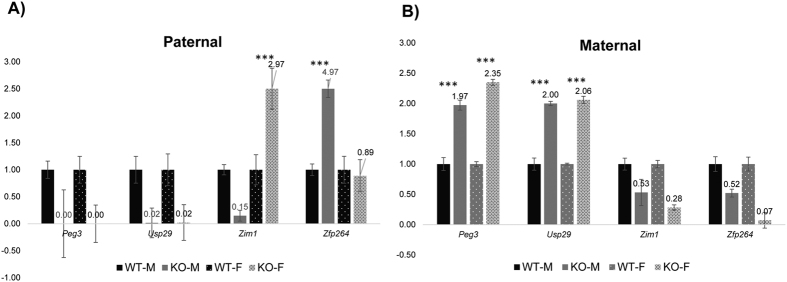
Mutational effects on the expression levels of the *Peg3* domain in neonates. A series of qRT-PCR were performed to test potential effects of the deletion on the transcriptional levels of the imprinted genes within the *Peg3* domain. This series of analyses used the total RNA isolated from the brain of the one-day-old male and female pups carrying the deleted allele paternally (**A**) and maternally (**B**). The expression levels of each gene were first normalized with an internal control (*Gapdh*), and the normalized values were further compared between the wild-types (WT) and the heterozygotes (KO). The relative levels are presented in a graph with standard errors (S.E.). The statistical significance of the observed differences between WT and KO was also tested with student t-test (*<0.05; **<0.01; ***<0.001). This series of analyses were repeated with two independent sets of biological replicates.

The mutational effects on the expression levels of the individual genes were also analyzed using the total RNA isolated from the neonatal brains and adult tissues that were harvested from the mutant mice. This series of expression analyses mainly targeted four genes (*Peg3, Usp29, Zim1, Zfp264*) since the expression levels of the other genes (*Zim2* and *Zim3*) are very low in both neonatal brains and adult tissues. The results are summarized as follows. First, the expression levels of both *Peg3* and *Usp29* were again undetectable in the brains of the neonates with the paternal transmission of the deletion (Fig. 4A). The expression levels of *Zim1* and *Zfp264*, on the other hand, were affected, but in a sex-specific manner. In the case of *Zim1*, the paternal transmission caused the reduction of the expression levels in males but an increase in females. In the case of *Zfp264*, the paternal transmission resulted in an increase only in males. Second, the maternal transmission of the deletion derived very unexpected outcomes in the expression levels of both *Peg3* and *Usp29* (Fig. 4B). In the female set, the expression levels of both *Peg3* and *Usp29* were 2-fold higher in the KO than in WT samples. In the male set, both genes also showed the increase but the observed increases were slightly lower than those from the female set. The observed up-regulation of both paternally expressed *Peg3* and *Usp29* was unexpected since the mutation causing the deletion of the bidirectional promoter was on the opposite maternal allele. In retrospect, however, this outcome is consistent with higher survival rates and larger body sizes of the neonatal mutants with the maternal transmission compared to their littermates (Fig. 2). It is likely that the increased levels of *Peg3* and *Usp29* are directly related to the increased survival and growth rates.

**Figure 5:**
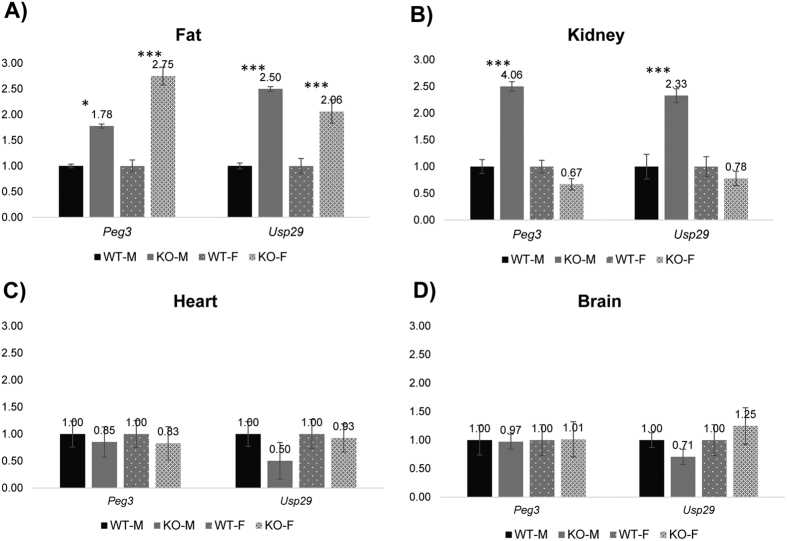
Mutational effects on the expression levels of the *Peg3* domain in adult tissues. A series of qRT-PCR were performed to test potential effects of the deletion on the transcriptional levels of the imprinted genes within the *Peg3* domain. This series of analyses used the total RNA isolated from adult tissues, including fat, kidney, heart, and brain of two-month-old male and female mice with the maternal transmission of the deleted allele. The expression levels of each gene were first normalized with an internal control (*Gapdh*), and the normalized values were further compared between the wild-types (WT) and the heterozygotes (KO). The relative levels are presented in a graph with standard errors (S.E.). The statistical significance of the observed differences between WT and KO was also tested with student t-test (*<0.05; **<0.01; ***<0.0001). This series of analyses were repeated with two independent sets of biological replicates.

Third, the results from the adult tissues also showed similar unexpected outcomes in the maternal transmission of the deletion (Fig. 5A,B). In the fat tissue, both *Peg3* and *Usp29* showed the increased expression levels in both sexes, but with different levels: 1.78-fold (male) versus 2.75-fold (female) for *Peg3* and 2.50-fold (male) versus 2.06-fold (female) for *Usp29*. In the kidney, both *Peg3* and *Usp29* showed increased expression levels only in males, but no statistically significant changes in females. In contrast, the expression levels of *Peg3* and *Usp29* were overall similar between WT and KO in the two remaining tissues, brain and heart (Fig. 5C,D). The expression levels of the two remaining genes, *Zim1* and *Zfp264*, were relatively low in adult-stage tissues, thus omitted in this series of expression analyses. Taken together, this series of analyses concluded that the paternal transmission of the deletion resulted in changes in the expression levels of the two adjacent genes, *Zim1* and *Zfp264*. Interestingly, the maternal transmission caused the unexpected up-regulation of the remaining paternal allele of two genes, *Peg3* and *Usp29*. The observed changes in both transmissions were also different between two sexes, suggesting that the transcription of the *Peg3* domain may be subject to unknown regulatory mechanisms involving sexual differentiation.

### Sexually biased lethality associated with the deletion of the Peg3-DMR

**Figure 6:**
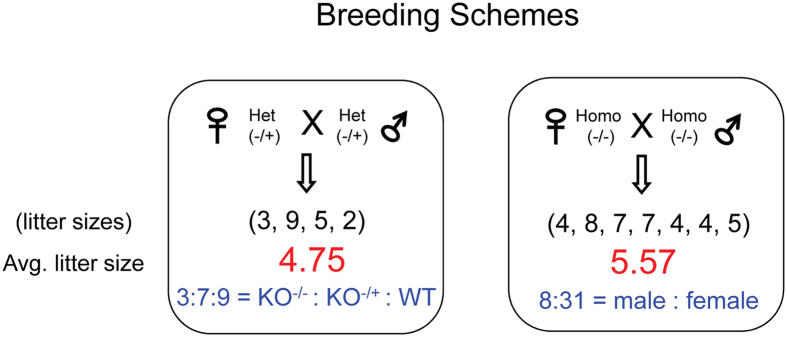
Sexually biased lethality associated with the deletion of the Peg3-DMR. Male and female heterozygotes inheriting the deleted allele maternally (−/+) were bred with each other for the derivation of homozygotes. The average litter sizes and the transmission ratios of the deleted allele (WT versus KO) were summarized and presented. The numbers inside parentheses indicate the sizes of individual litters. In the case of homozygote breeding, the sex ratio was presented.

The potential lethality associated with the homozygous deletion of the Peg3-DMR was further tested using the following breeding scheme (Fig. 6). First, the male and female heterozygotes for the deletion were bred together for the derivation of the homozygotes. This intercrossing of the two heterozygotes derived a much smaller litter size (4.75) with the skewed transmission ratio (KO^−/−^:KO^−/+^:WT^+/+^ = 3:7:9) (chi-square test, *p* = 0.0778), suggesting the potential embryonic lethality associated with the homozygotes and also possibly heterozygotes. Given a small number of litters tested so far, this needs to be further confirmed through additional breeding experiments. The three obtained homozygotes, one male and two females, were again used for the additional breeding. In this case, the average litter size was 5.57, but with a very skewed sex ratio (male:female = 8:31) (chi-square test, *p* = 0.00004), suggesting that the deletion of the Peg3-DMR affects more severely the survival of the males than the females. Overall, this series of breeding experiments concluded that the homozygotes are lethal during early embryonic stages, and that the males tend to be more severely affected than the females, another sex-biased effect associated with the *Peg3* domain.

## Discussion

**Figure 7:**
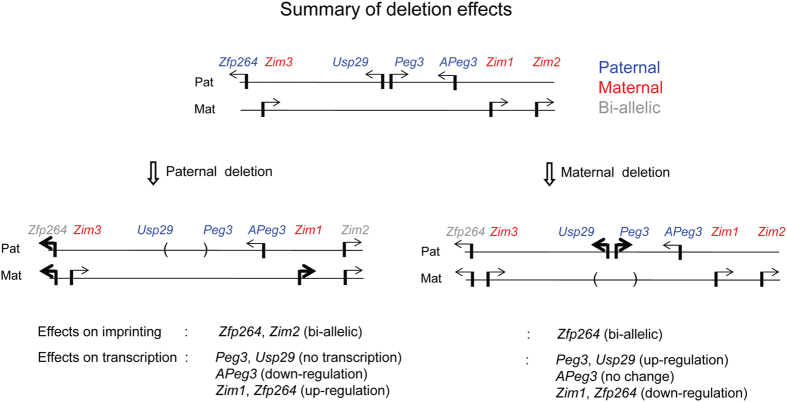
Summary of the deletion effects on the imprinting and expression of the *Peg3* domain. A schematic representation of the *Peg3* domain is shown on the upper panel. Each imprinted gene is indicated with an arrow. The paternally and maternally expressed genes are indicated with blue and red, respectively. The bi-allelically expressed genes are indicated with gray. The deletion of the Peg3-DMR is indicated with a pair of parentheses. The mutational effects by the deletion of the Peg3-DMR with the paternal and maternal transmission are summarized with two schematic diagrams on the bottom panels. The mutational effects on the imprinting status were summarized using the results derived from the total RNA that had been isolated from the neonatal brains of F1 bybrids with both sexes. The mutational effects on expression levels were also summarized using the results derived from the total RNA that had been isolated from the neonatal brains with both sexes. The deletion tends to have similar effects on the expression levels between two sexes except *Zim1*, in which the paternal transmission has two different effects: down-regulation in males versus up-regulation in females.

In the current study, we characterized the deletion effects of the Peg3-DMR on the expression levels of the *Peg3* domain and also on the survival and growth rates of the animals. As expected, the paternal transmission of the deletion of this ICR resulted in global effects on the expression levels as well as the imprinting status of the *Peg3* domain, reconfirming the ICR roles played by the Peg3-DMR (Fig. 7). The maternal transmission, on the other hand, derived very unexpected outcomes: the increased expression levels of the remaining paternal alleles of *Peg3* and *Usp29* and the increased survival and growth rates among the mutant animals. This suggests that the imprinted maternal allele of the Peg3-DMR may function as a suppressor negatively influencing the remaining paternal allele of the ICR.

One of the unexpected outcomes from the current study is the phenotypic effect caused by the deletion of the maternal allele of the Peg3-DMR, although the maternal allele of this ICR is presumably silenced due to DNA methylation. Breeding experiments demonstrated that the animals lacking the maternal allele were more survivable and also bigger than their littermates with the normal methylated allele (Fig. 2). This is also the case for the expression levels of *Peg3* and *Usp29* with both genes showing higher levels in the animals lacking the maternal allele than in the animals with the methylated allele (Figs 4 and 5). This is quite different from the outcome from the other ICRs that the deletion of the repressed (or imprinted) allele of ICRs usually causes no obvious phenotypic or expression differences compared to their wild-type littermates^21^,^22^,^23^. One of key interpretations from these unusual observations is that the methylated allele of the Peg3-DMR might serve as a functional suppressor antagonistic to the remaining active allele. As such, the deletion of this suppressor might cause the up-regulation of *Peg3* and *Usp29*, and the corresponding increase in the survival and growth rates of the animals. This is purely conceptual at the moment without any mechanistic details, but it might be feasible in both direct and indirect ways. First, the deletion of the methylated ICR might change the chromatin structure and concurrent expression levels of the adjacent imprinted genes, causing changes in the expression levels of the independent loci that might be crucial for the transcriptional control of *Peg3* and *Usp29*. Through unknown loci, the maternal allele may end up indirectly controlling the paternal allele. Second, the deletion of the methylated ICR might directly impact the active paternal allele through either transvection or paramutation-like mechanisms, which have been seen in insects and plants, respectively^24^,^25^. It is relevant to note that this trans-allelic phenomenon has also been observed in the other imprinted domains, including *Snrpn/Ube3a*^26^, *Rasgrf1*^27^,^28^, and *Gtl2/Dlk1* domains^29^. As shown in the transvection mode, one possibility might be that the two alleles of the Peg3-DMR share, trans-allelically, some of the enhancers that are located within this imprinted domain^30^,^31^. In this scenario, deleting one competing allele of the Peg3-DMR might be a boon for the remaining allele, thus increasing the expression levels of *Peg3* and *Usp29*. Overall, characterizing the unknown mechanistic aspects of this trans-allelic regulation should be an interesting avenue for the future study on the *Peg3* domain.

**Figure 8:**
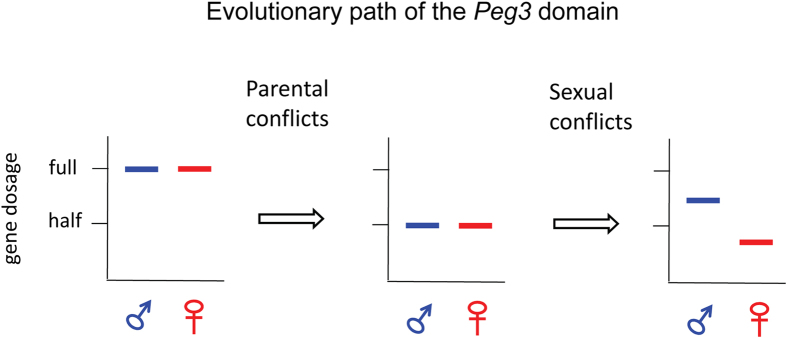
Schematic diagram for parental and sexual conflict over the *Peg3* domain. The *Peg3* domain may be subject to two different regulatory mechanisms: genomic imprinting to resolve parental conflicts and another unknown mechanism to resolve sexual conflicts. The three graphs illustrate the hypothetical evolutionary path for the *Peg3* domain. The ancestral locus may have started with the bi-allelic expression of the *Peg3* domain (left); the parental conflicts may be later responsible for down-regulating the gene dosage of the *Peg3* domain from the full to half dosage (middle); and finally the sexual conflict may have further up- or down-regulated the half-dosage of the *Peg3* domain based on the opposite needs from both sexes (right).

The deletion of the Peg3-DMR tends to derive sexually biased effects: the observed up-regulation of *Peg3* and *Usp29* is more dramatic in females than in males (Figs 4 and 5). Also, the breeding experiments indicated that the loss of the *Peg3* expression causes more severe impact on males than on females (Fig. 6). These sexually biased outcomes might be explained in the following manner. The *Peg3* domain may be subject to two different regulatory mechanisms: genomic imprinting to resolve parental conflicts and an uncharacterized mechanism to resolve sexual conflicts (Fig. 8). Genomic imprinting is responsible for the repression of the maternal allele through DNA methylation during oogenesis, and the subsequent mono-allelic expression (or half dosage) of both *Peg3* and *Usp29* in somatic cells. The potential mechanism resolving sexual conflicts, on the other hand, might further modulate the remaining half dosage, but in opposite directions between two sexes. The remaining paternal allele might be further up-regulated in males, whereas further down-regulated in females, resulting in sexually-biased expression levels. This has been indeed demonstrated through the previous study showing higher levels of *Peg3* expression in males than in females, particularly during late embryonic and perinatal stages^10^. The different levels of *Peg3* between two sexes might have been established to satisfy the opposite needs from both sexes. Since *Peg3* is known to be important for muscle differentiation^8^,^32^,^33^, higher levels of the *Peg3* expression might be beneficial for males, which require a large amount of muscle mass but a relatively small amount of fat mass. In contrast, lower levels of the *Peg3* expression might be better for females since the energy could be saved for a large amount of fat mass at the expense of muscle mass, which might be needed for the reproduction and nursing functions of females. This also has been demonstrated by the mutant phenotypes of *Peg3* that the mutant mice tend to have a reduced amount of muscle mass but an increased amount of fat mass^34^. Since the expression levels of *Peg3* are already set up at two different levels between sexes, higher in males and lower in females, complete abolition of the *Peg3* expression might also have different levels of functional severity between two sexes: much greater levels of the down-regulation and concurrent impact on males than on females. This might be the reason for the missing of males in the homozygote breeding experiment (Fig. 6). If this is indeed the case, the bi-allelic expression of *Peg3* might be more tolerable for males but very detrimental for females, since males are already optimized for the higher levels of the *Peg3* expression. On a separate note, the proposed sexual conflict might have been a main driver conferring a suppressor role to the maternal allele of the Peg3-DMR to antagonize the paternal allele. A similar situation, also known as intralocus sexual conflict, has been often observed from the genetic loci of the other taxa, such as insects, which have conflicting roles between two sexes^35^,^36^. In conclusion, we need to perform more analyses to test whether this model invoking two different conflicts is the correct explanation for the unusual outcomes described in the current study. Nevertheless, it is very likely that the *Peg3* domain is controlled through several mechanisms other than genomic imprinting.

## Materials and Methods

### Ethics statement

All the experiments related to mice were performed in accordance with National Institutes of Health guidelines for care and use of animals, and also approved by the Louisiana State University Institutional Animal Care and Use Committee (IACUC), protocol #13–061.

### Mouse strain and breeding

For the current study, the targeted allele with two loxP sites flanking the 4-kb Peg3-DMR has been generated through knockout experiments as described previously^19^. The mouse strain containing this floxed allele was bred with the Zp3-Cre line (Jackson lab, Stock No. 003651), subsequently generating the mutant strain lacking the 4-kb Peg3-DMR. This mutant strain with the deleted allele was used for breeding experiments in this study. The pups generated from various breeding schemes were analyzed in terms of their sex, genotype and health status. The sex and genotype were determined through PCR using the following two primer sets: mSry-F (5′-GTCCCGTGGTGAGAGGCACAAG-3′) and mSry-R (5′-GCAGCTCTACTCCAGTCTTGCC-3′) for the sex and Primer A (5′-TGACAAGTGGGCTTGCTGCAG-3′), B (5′-GGATGTAAGATGGAGGCACTGT-3′), C (5′-ACAACCCGGAGTTTTAGCAGAC-3′), and D (5′-AGGGGAGAACAGACTACAGA-3′) for the genotype. The genomic DNA was isolated from tail snips through incubating the tissue samples at 55° C in the following lysis buffer overnight (0.1 M Tris-Cl, pH 8.8, 5 mM EDTA, pH 8.0, 0.2% SDS, 0.2 M NaCl, 20 μg/ml Proteinase K). For southern blotting, the genomic DNA were isolated from WT and KO animals, digested with *BamH*I, transferred onto a nylon membrane, and hybridized with a probe derived from the genomic region (mm10, chr7: 6,717,796-6,718,290), which is outside of the 3′-side homology hook for the targeting construct.

### Imprinting tests

For the imprinting tests, the mutant strain of the 129/B6 background was reciprocally crossed with the PWD/PhJ strain (Jackson Lab, Stock No. 004660). The brains of the one-day-old neonates of this F1 hybrid were used for isolating total RNA, which was subsequently used for synthesizing cDNA for RT-PCR-based imprinting tests. The amplified PCR product for each imprinted gene was digested with a given enzyme to visualize the ratio between two types of the DNA fragments representing two parental alleles. Detailed information regarding the polymorphism and corresponding restriction enzyme for each gene is available upon request and also through the previous study^11^.

### DNA methylation analyses

For DNA methylation analyses, genomic DNA from neonatal brains was treated with the bisulfite conversion protocol^37^. The converted DNA was then used as a template for PCR to amplify each target region. The amplified products were analyzed with COBRA (COmbined Bisulfite and Restriction Analysis)^38^. The information regarding the sequences of oligonucleotides and the PCR conditions for each genomic region is available through the previous study^11^.

### Expression analyses

For the expression analyses, total RNA was isolated from the heads of one-day-old neonates and adult tissues of two-month-old age using a commercial kit (Trizol, Invitrogen). The isolated RNA was reverse-transcribed using the M-MLV kit (Invitrogen), and the subsequent cDNA was used for quantitative RT-PCR analyses. This analysis was performed with the iQ SYBR green supermix (Bio-Rad) using the *ViiA™ 7 Real-Time PCR* System (*Life Technologies*). All qRT-PCR reactions were carried out for 40 cycles under standard PCR conditions. The results were analyzed using the double delta Ct method as described previously^39^. An internal control (*Gapdh*) was mainly used for this series of expression analyses. The detailed information regarding the primers and PCR condition for each gene is available upon request and also available through the previous study^11^.

## Additional Information

**How to cite this article**: He, H. *et al*. Parental and sexual conflicts over the *Peg3* imprinted domain. *Sci. Rep.* **6**, 38136; doi: 10.1038/srep38136 (2016).

**Publisher's note:** Springer Nature remains neutral with regard to jurisdictional claims in published maps and institutional affiliations.

## Supplementary Material

Supplementary Information
