# Supplementary material for: Parental and sexual conflicts over the Peg3 imprinted domain
Source: Sci Rep. 2016 Nov 30;6:38136. doi: 10.1038/srep38136 (PMC5128876; doi:10.1038/srep38136)
Supplement: Supplementary Information [file srep38136-s1.pdf]

**Supplementary Materials**

# Parental and sexual conflicts over the *Peg3* imprinted domain

Hongzhi He, Bambarendage P.U. Perera, An Ye, Joomyeong Kim

Supplemental Material 1

## Fig2 A

### Paternal transmission

| ID No | Sex | raw weight | Genotype | % |
|---|---|---|---|---|
| 112 | M | 1.13 | 0 | 0.753 |
| 144 | F | 1.17 | 0 | 0.813 |
| 145 | F | 1.19 | 0 | 0.826 |
| 122 | M | 1.45 | 0 | 0.843 |
| 117 | F | 1.27 | 0 | 0.847 |
| 113 | M | 1.27 | 0 | 0.847 |
| 167 | F | 1.43 | 0 | 0.851 |
| 164 | M | 1.43 | 1 | 0.851 |
| 181 | F | 1.28 | 0 | 0.853 |
| 175 | M | 1.33 | 0 | 0.887 |
| 141 | M | 1.28 | 0 | 0.889 |
| 183 | F | 1.35 | 0 | 0.900 |
| 116 | F | 1.36 | 0 | 0.907 |
| 121 | M | 1.56 | 0 | 0.907 |
| 169 | F | 1.53 | 0 | 0.911 |
| 111 | M | 1.37 | 0 | 0.913 |
| 179 | F | 1.41 | 1 | 0.940 |
| 131 | F | 1.63 | 1 | 0.948 |
| 101 | M | 1.34 | 0 | 0.950 |
| 142 | M | 1.37 | 0 | 0.951 |
| 162 | M | 1.6 | 1 | 0.952 |
| 126 | F | 1.64 | 0 | 0.953 |
| 174 | M | 1.44 | 1 | 0.960 |
| 107 | F | 1.36 | 0 | 0.965 |
| 147 | F | 1.39 | 0 | 0.965 |
| 125 | M | 1.67 | 0 | 0.971 |
| 102 | F | 1.37 | 1 | 0.972 |
| 182 | F | 1.46 | 1 | 0.973 |
| 173 | M | 1.47 | 0 | 0.980 |
| 177 | M | 1.47 | 0 | 0.980 |
| 104 | F | 1.42 | 0 | 1.007 |
| 103 | F | 1.42 | 1 | 1.007 |
| 129 | F | 1.74 | 0 | 1.012 |
| 128 | F | 1.75 | 1 | 1.017 |
| 124 | M | 1.75 | 1 | 1.017 |
| 146 | F | 1.47 | 0 | 1.021 |
| 165 | M | 1.72 | 1 | 1.024 |
| 105 | F | 1.45 | 0 | 1.028 |
| 123 | M | 1.8 | 1 | 1.047 |
| 118 | F | 1.58 | 1 | 1.053 |
| 143 | M | 1.52 | 0 | 1.056 |
| 171 | M | 1.6 | 1 | 1.067 |
| 163 | M | 1.8 | 1 | 1.071 |
| 172 | M | 1.61 | 1 | 1.073 |
| 106 | F | 1.53 | 1 | 1.085 |
| 166 | F | 1.83 | 1 | 1.089 |
| 148 | F | 1.59 | 1 | 1.104 |
| 168 | F | 1.86 | 1 | 1.107 |
| 176 | M | 1.67 | 1 | 1.113 |
| 130 | F | 1.92 | 1 | 1.116 |
| 149 | F | 1.61 | 1 | 1.118 |
| 150 | F | 1.62 | 1 | 1.125 |
| 127 | F | 1.97 | 1 | 1.145 |
| 151 | F | 1.65 | 1 | 1.146 |
| 180 | F | 1.74 | 1 | 1.160 |
| 161 | M | 1.96 | 1 | 1.167 |

## Fig 2 B

### Maternal transmission

WT=1, KO=0

| ID No | Sex | raw weight | Genotype | % |
|---|---|---|---|---|
| 211 | M | 2.02 | 0 | 0.808 |
| 205 | F | 1.26 | 0 | 0.818 |
| 246 | F | 1.28 | 0 | 0.859 |
| 222 | F | 1.37 | 0 | 0.895 |
| 247 | F | 1.35 | 0 | 0.906 |
| 202 | M | 1.41 | 1 | 0.916 |
| 236 | F | 1.53 | 0 | 0.916 |
| 217 | F | 2.35 | 0 | 0.940 |
| 234 | M | 1.59 | 0 | 0.952 |
| 237 | F | 1.59 | 1 | 0.952 |
| 244 | F | 1.42 | 0 | 0.953 |
| 226 | M | 1.46 | 0 | 0.954 |
| 228 | M | 1.46 | 0 | 0.954 |
| 233 | M | 1.6 | 0 | 0.958 |
| 214 | F | 2.41 | 1 | 0.964 |
| 248 | F | 1.44 | 0 | 0.966 |
| 229 | M | 1.48 | 0 | 0.967 |
| 218 | F | 2.45 | 0 | 0.980 |
| 238 | F | 1.64 | 1 | 0.982 |
| 213 | F | 2.46 | 1 | 0.984 |
| 221 | F | 1.51 | 1 | 0.987 |
| 204 | M | 1.54 | 1 | 1.000 |
| 225 | M | 1.54 | 1 | 1.007 |
| 208 | F | 1.57 | 1 | 1.019 |
| 223 | F | 1.56 | 0 | 1.020 |
| 227 | M | 1.56 | 0 | 1.020 |
| 219 | F | 2.55 | 1 | 1.020 |
| 232 | M | 1.71 | 0 | 1.024 |
| 201 | M | 1.58 | 0 | 1.026 |
| 206 | F | 1.59 | 0 | 1.032 |
| 243 | M | 1.55 | 0 | 1.040 |
| 245 | F | 1.55 | 1 | 1.040 |
| 212 | M | 2.61 | 1 | 1.044 |
| 207 | F | 1.63 | 0 | 1.058 |
| 216 | F | 2.66 | 0 | 1.064 |
| 220 | F | 2.7 | 0 | 1.080 |
| 235 | M | 1.81 | 0 | 1.084 |
| 230 | M | 1.66 | 1 | 1.085 |
| 215 | F | 2.73 | 1 | 1.092 |
| 224 | F | 1.69 | 0 | 1.105 |
| 242 | M | 1.67 | 1 | 1.121 |
| 241 | M | 1.68 | 0 | 1.128 |
| 203 | M | 1.74 | 1 | 1.130 |
| 231 | M | 1.92 | 1 | 1.150 |

| | | | | |
|---|---|---|---|---|
| 178 | M | 1.75 | 1 | 1.167 |
| 119 | F | 1.76 | 1 | 1.173 |
| 120 | F | 1.8 | 1 | 1.200 |
| 114 | M | 2.02 | 1 | 1.347 |

## Fig2 C

### Paternal transmission

| ID No | Sex | raw weight | Genotype | % |
|---|---|---|---|---|
| M1 | M | 26 | 1 | 1.227 |
| M2 | M | 26 | 1 | 1.227 |
| M3 | M | 29 | 1 | 1.369 |
| M4 | M | 23 | 1 | 1.086 |
| M5 | M | 23 | 1 | 1.086 |
| F6 | F | 19 | 1 | 0.897 |
| F7 | F | 19 | 1 | 0.897 |
| F8 | F | 19 | 1 | 0.897 |
| M6 | M | 22 | 0 | 1.038 |
| M7 | M | 23 | 0 | 1.086 |
| M8 | M | 20 | 0 | 0.944 |
| F1 | F | 17 | 0 | 0.802 |
| F2 | F | 17 | 0 | 0.802 |
| F3 | F | 17 | 0 | 0.802 |
| F4 | F | 16 | 0 | 0.755 |
| F5 | F | 23 | 1 | 1.086 |
| M9 | M | 29 | 1 | 1.192 |
| M10 | M | 25 | 0 | 1.027 |
| M11 | M | 19 | 0 | 0.781 |

## Fig2 D

### Paternal transmission

| ID No | Sex | raw weight | Genotype | % |
|---|---|---|---|---|
| M1 | M | 21 | 1 | 1.026 |
| M2 | M | 19 | 1 | 0.929 |
| M3 | M | 20 | 1 | 0.978 |
| M4 | M | 23 | 0 | 1.124 |
| M5 | M | 26 | 0 | 1.271 |
| M6 | M | 26 | 0 | 1.271 |
| F1 | F | 19 | 1 | 0.929 |
| F2 | F | 17 | 1 | 0.831 |
| F3 | F | 21 | 0 | 1.026 |
| F4 | F | 21 | 0 | 1.026 |
| F5 | F | 19 | 1 | 0.929 |
| F6 | F | 18 | 1 | 0.88 |
| F7 | F | 16 | 1 | 0.782 |
| M7 | M | 32 | 0 | 1.185 |
| M8 | M | 28 | 1 | 1.037 |
| M9 | M | 26 | 1 | 0.963 |
| M10 | M | 28 | 0 | 1.037 |
| F8 | F | 26 | 0 | 0.963 |
| F9 | F | 26 | 1 | 0.963 |
| F10 | F | 26 | 0 | 0.963 |
| F11 | F | 24 | 1 | 0.889 |
| M11 | M | 9.8 | 0 | 1.018 |
| M12 | M | 10.8 | 0 | 1.122 |
| M13 | M | 9.1 | 1 | 0.946 |
| F12 | F | 8.2 | 1 | 0.852 |
| F13 | F | 8.6 | 1 | 0.894 |
| F14 | F | 10.7 | 0 | 1.112 |
| F15 | F | 9.4 | 0 | 0.977 |
| F16 | F | 9.7 | 1 | 1.008 |
| F17 | F | 10.3 | 0 | 1.07 |

Supplemental_material2_He

Imprinting Test with Female sets

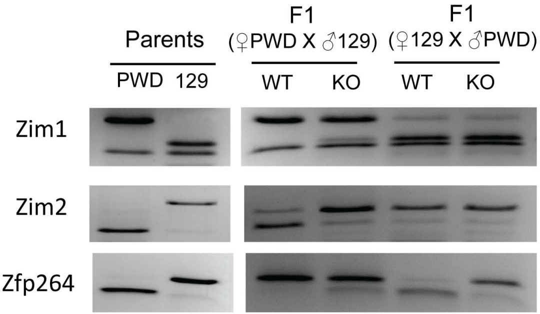


**Supplemental material 2**. Imprinting tests using female hybrid sets. The male set is shown in Figure 3, and detailed annotations are same as Figure 3.

Supplemental_material3_He

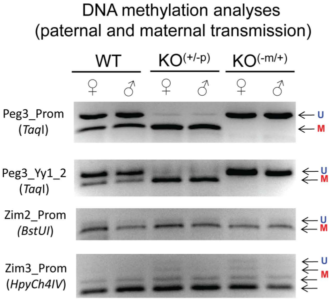


**Supplemental material 3**. DNA methylation analyses using the DNA isolated from the neonatal brains of wild type (WT) and knockout (KO) mice with the paternal and maternal transmission of the mutant allele. The bisulfite-converted DNA was amplified with primer sets targeting Peg3-DMR (Peg3_Prom, Peg3_Yy1_2), Zim2-DMR, and Zim3-DMR. The amplified products were digested with individual enzymes that can determine the original methylation status of the surveyed DNA. Overall, the Zim3-DMR was affected only in the female set of the KO animals, showing partially digested fragments.
